# Spontaneous Entero‐Scrotal Fistula: A Rare Complication of Inguinal Hernia in a Developed Country—A Case Report

**DOI:** 10.1155/cris/9362137

**Published:** 2026-05-30

**Authors:** Immanuella Owusu-Ansa, Femi E. Ayeni, Daniel Vagg, Senarath Edirimanne

**Affiliations:** ^1^ Department of Surgery, Nepean Hospital, Penrith, New South Wales, Australia, nbmlhd.health.nsw.gov.au; ^2^ Faculty of Medicine and Health, The University of Sydney, Camperdown, New South Wales, Australia, sydney.edu.au; ^3^ Nepean Institute of Academic Surgery, The University of Sydney, Penrith, New South Wales, Australia, sydney.edu.au

## Abstract

**Introduction and Importance:**

Entero‐scrotal fistula is an exceptionally rare and severe complication of inguinal hernias in adults. We present the first reported Australian case, highlighting the unique diagnostic challenges of Richter‐type hernias, particularly in socially isolated patients, where presentation is often delayed.

**Case Presentation:**

A 71‐year‐old male with a history of hypertension and chronic alcohol disorder was transferred from a group home with sepsis and a necrotic scrotal ulcer. Computed tomography confirmed a right inguinal hernia with an enterocutaneous fistula originating from the terminal ileum. Intraoperative findings revealed a Richter’s hernia with the antimesenteric wall of the terminal ileum incarcerated within the deep inguinal ring, leading to a spontaneous fistula through the scrotal wall. Management involved an ileocecal resection with side‐to‐side anastomosis and debridement of necrotic scrotal tissue. Due to gross fecal contamination, a primary tissue repair was performed rather than mesh placement.

**Clinical Discussion:**

Spontaneous fistulization typically results from prolonged incarceration. In this case, the Richter‐type hernia allowed for intermittent bowel patency, which masked typical obstructive symptoms and delayed surgical intervention. In the setting of gross contamination (Type IV), primary suture repair—such as the “Nylon Darn” technique—is preferred over synthetic mesh to prevent prosthetic infection and chronic wound complications.

**Conclusion:**

This case underscores the importance of considering socioeconomic factors and atypical hernia types in surgical delays. In contaminated fields, primary tissue repair remains the gold standard to ensure a safe recovery and optimal patient outcomes.

## 1. Introduction

An inguinal hernia is the protrusion of abdominal contents, typically fat or bowel, into the inguinal canal [1]. While hernias are among the most common surgical pathologies globally, they can present with life‐threatening complications such as obstruction, incarceration, and strangulation [2]. Rare variants include an Amyand’s hernia—where the appendix is found within the hernial sac—which occurs in ~1% of cases [3]. Of clinical significance is the Richter‐type hernia, characterized by the entrapment of only a portion of the bowel wall circumference, typically the antimesenteric border. This anatomy is notoriously deceptive because bowel continuity is maintained, patients often lack the classic signs of high‐grade intestinal obstruction, such as absolute constipation or significant abdominal distension [4]. Consequently, diagnosis is frequently delayed, potentially leading to severe sequelae such as spontaneous entero‐scrotal fistulization.

Spontaneous entero‐scrotal fistula resulting from prolonged hernia incarceration is an exceptionally rare complication in adults [5–10]. Paradoxically, fistula formation may create a point of decompression, thereby obscuring the underlying pathology and further delaying diagnosis and surgical intervention. This case is noteworthy as it illustrates how Richter‐type hernias can progress to advanced complications in the absence of classic obstructive symptoms, particularly among patients facing social barriers to accessing healthcare. To our knowledge, this represents the first documented case of an entero‐scrotal fistula in Australia. This report has been prepared in accordance with the SCARE 2023 guidelines [11].

## 2. Case Presentation

**Figure 1:**
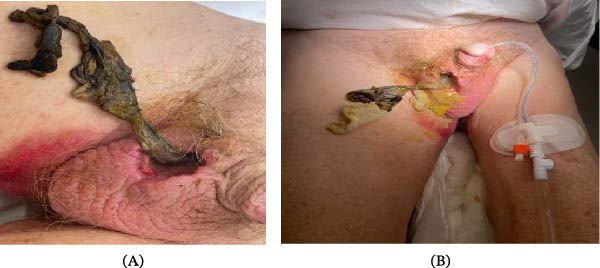
(A) Close‐up clinical photograph of the right hemiscrotum demonstrating marked erythema and drainage of feculent material through a necrotic scrotal defect. (B) Wide‐angle view illustrating the fistula in the context of the patient’s general habitus, with associated areas of skin necrosis.

A 71‐year‐old Caucasian male with a history of hypertension and chronic alcohol use disorder, residing in an independent group home, was transferred to our tertiary center with a presumed scrotal ulcer and systemic sepsis. The patient had initially presented to the emergency department of a regional hospital with generalized malaise, along with fecal and urinary incontinence. On examination, the patient was tachycardic (117 bpm), Hypotensive (95/50 mmHg) and febrile (38.1°C). The right hemiscrotum was significantly erythematous, and there was obvious necrosis of the right hemiscrotum with feculent material (Figure 1A,B). Notably, bowel sounds were audible upon auscultation of the scrotum, leading to a provisional diagnosis of enteroscrotal fistula.

**Figure 2:**
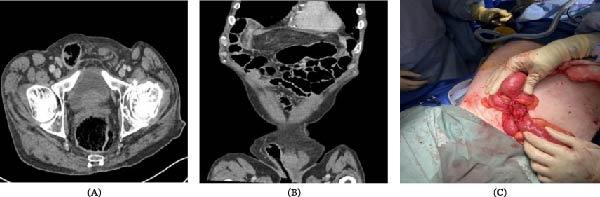
(A) Axial and (B) coronal CT images of the abdomen and pelvis demonstrating a right inguinal hernia containing small bowel loops, with associated inflammatory changes and gas tracking into the scrotal sac. (C) Intraoperative view showing an incarcerated and perforated Richter‐type hernia at the internal inguinal ring, involving the antimesenteric border of the terminal ileum.

Diagnostic imaging, both axial (Figure 2A) and coronal (Figure 2B) computed tomography scans, both demonstrate a right inguinal hernia with resultant entero‐cutaneous fistula communicating with the scrotal skin arising from the terminal ileum. Laboratory results revealed a hemoglobin of 125 g/L and a white cell count of 10.0 × 109/L. Inflammatory markers were elevated with a C‐reactive protein of 113 mg/L. Electrolyte analysis showed hyponatremia with a sodium of 126 mmol/L, mild hypokalemia at 3.4 mmol/L, a magnesium of 0.53 mmol/L, and a phosphate of 0.56 mmol/L. The venous pH was 7.3. Microbiological analysis of both tissue and wound swabs cultured *Enterobacter cloacae* and *Parabacteroides distasonis*.

The patient was stabilized with intravenous fluid resuscitation and broad‐spectrum antibiotics and taken to the operating theater for emergent exploration under general anesthesia by a joint urology and general surgery team. A standard inguinal incision was made ~1 cm above and parallel to the inguinal ligament. Intraoperative findings revealed a Richter‐type hernia, with the antimesenteric border of the terminal ileum incarcerated and perforated at the deep inguinal ring (Figure 2C). Gross fecal contamination was present throughout the inguinal canal and scrotum (Type IV contamination) (Figure 1B). Due to the degree of bowel compromise, a midline laparotomy was performed to facilitate an ileocecal resection with a side‐to‐side functional end‐to‐end anastomosis. Extensive debridement of the necrotic scrotal skin and the right testicle was required. The testicular necrosis was attributed to prolonged mechanical compression of the spermatic cord at the narrow hernia neck, leading to venous outflow obstruction of the pampiniform plexus and subsequent ischemic infarction. Given the gross contamination, synthetic mesh was contraindicated. The hernia was managed with a primary tissue repair using the Nylon Darn (Moloney) technique. This involved a continuous, tension‐free weave of monofilament nylon between the conjoint tendon and the shelving edge of the inguinal ligament, extending from the pubic tubercle to the internal ring.

The scrotal wound was left open to heal by secondary intention, with regular dressing changes. Postoperative recovery was uneventful, and the patient was discharged to his care facility on postoperative day 10. At 6‐month follow‐up, the scrotal wound had completely epithelialized, with no evidence of hernia recurrence.

## 3. Discussion

An inguinal hernia is the protrusion of abdominal contents, most commonly preperitoneal fat or bowel, into the inguinal canal [1]. During fetal development, the testes descend from the posterior abdominal wall into the scrotum, creating a natural area of weakness along the processus vaginalis. An indirect inguinal hernia follows this embryological pathway through the internal inguinal ring, potentially allowing bowel to descend into the scrotum [2, 3]. While most inguinal hernias are readily manageable, prolonged incarceration may result in rare, life‐threatening complications. Among the various subtypes, Richter’s hernia is characterized by herniation of only the antimesenteric border of the bowel wall through the defect [5].

This variant is notoriously deceptive, as it may not cause complete bowel obstruction, leading to significant diagnostic delay. If left untreated, strangulation results in bowel ischemia with subsequent transmural necrosis and tissue loss. In extreme cases, this process extends to surrounding structures—including the spermatic cord and scrotal wall—resulting in a direct communication between the bowel lumen and the skin, forming a spontaneous entero‐scrotal fistula [6–8].

Spontaneous entero‐scrotal fistula is an exceptionally rare complication in adults; prior to a landmark report in 2005 [7], it had been reported only in infants. A comprehensive literature review (PubMed, EBSCO, and MEDLINE) identified only 12 reported adult cases worldwide, predominantly from under‐resourced countries (e.g., India, Ghana, and Nigeria [8–11]). While cost barriers and limited access to healthcare are major contributing factors in these settings, this case, along with the two cases reported in the United States [6], demonstrates that social isolation and limited individual health literacy can result in similarly advanced surgical presentations even within well‐resourced healthcare systems.

The management of incarcerated or strangulated inguinal hernias requires careful intraoperative judgment, with the choice of repair guided primarily by bowel viability and the degree of surgical field contamination [12]. Mesh repair is generally appropriate when the bowel is viable or when strangulation resolves without gross contamination, including selected cases involving bowel resection in a clean or clean‐contaminated field. However, in the presence of bowel perforation with faeculent contamination, the use of synthetic polypropylene mesh is discouraged due to the substantially increased risk of surgical site infection, chronic sinus formation, and mesh sepsis. In such circumstances, suture‐based tissue repair techniques or the use of absorbable or biological mesh are recommended [13].

One established suture‐based method is the nylon darn (Moloney) technique, originally described by Abrahamson and later popularized by Moloney [14]. This technique involves approximating the conjoint tendon to the shelving edge of the inguinal ligament using a continuous, tension‐free monofilament suture, extending from the pubic tubercle laterally beyond the deep inguinal ring and then reversed medially to reinforce the posterior wall of the inguinal canal. The nylon darn repair is particularly useful in contaminated operative fields where prosthetic mesh placement is contraindicated.

## 4. Conclusion

This case represents the first reported Australian instance of a spontaneous entero‐scrotal fistula, a rare complication of Richter‐type hernia. It highlights how the absence of classic obstructive symptoms can delay diagnosis and result in severe tissue loss, even in developed healthcare settings. Prompt surgical management using a nylon darn repair achieved a favorable outcome. Clinicians should maintain a high index of suspicion in socially vulnerable patients to ensure timely intervention.

## Author Contributions

Conceptualization: Immanuella Owusu‐Ansa, Daniel Vagg, and Senarath Edirimanne. Collected consent and data: Immanuella Owusu‐Ansa. Writing – original draft: Immanuella Owusu‐Ansa, Femi E. Ayeni, Daniel Vagg, and Senarath Edirimanne. Writing – final manuscript edition: Immanuella Owusu‐Ansa, Femi E. Ayeni, Daniel Vagg, and Senarath Edirimanne.

## Funding

The authors have nothing to report. Open access publishing facilitated by The University of Sydney, as part of the Wiley ‐ The University of Sydney agreement via the Council of Australasian University Librarians.

## Disclosure

This case report was presented as a poster at the Royal Australasian College of Surgeons conference held in Christchurch, New Zealand, 2024.

## Ethics Statement

Approval from an institutional board review is not required for a case report. All the procedures performed in this study were in accordance with the ethical standards of the 1964 Helsinki Declarations. This report does not contain any animals performed by any of the authors.

## Consent

The patient provided the informed consent for the publication of this case report.

## Conflicts of Interest

The authors declare no conflicts of interest.

## Data Availability

The data that support the findings of this study are available upon request from the corresponding author. The data are not publicly available due to privacy or ethical restrictions.

## References

[1] Kingsnorth A. and LeBlanc K. , Management of Abdominal Hernias, The Lancet. (2003) 362, no. 9395, 1561–1571, 10.1016/S0140-6736(03)14746-0, 2-s2.0-0242403084. 14615114

[2] Solomon C. G. , Fitzgibbons R. J. , and Forse R. A. , Clinical Practice. Groin Hernias in Adults, The New England Journal of Medicine. (2015) 372, no. 8, 756–763, 10.1056/NEJMcp1404068, 2-s2.0-84922981710. 25693015

[3] D’Alia C. , Lo Schiavo M. G. , and Tonante A. , et al.Amyand’s Hernia: Case Report and Review of the Literature, Hernia. (2003) 7, no. 2, 89–91, 10.1007/s10029-002-0098-5, 2-s2.0-0038198660. 12820031

[4] Steinke W. and Zellweger R. , Richter’s Hernia and Sir Frederick Treves: An Original Clinical Experience, World Journal of Surgery. (2000) 24, no. 12, 1540–1545. 10.1097/00000658-200011000-00014 PMC1421226 11066144

[5] Samad A. , Spontaneous Scrotal Enterocutaneous Fistula as a Presentation of Inguinal Hernia, Journal of the College of Physicians and Surgeons Pakistan. (2005) 15, no. 10, 654–655.

[6] Perrotti M. , Smith M. J. , McGreevy C. J. , and Fischer J. R. , Spontaneous Enterocutaneous Fistula, The American Surgeon. (2022) 88, no. 7, 1653–1655.33629873

[7] Malik P. , Rathi M. , and Kumar K. , et al.Scrotal Enterocutaneous Fistula: A Rare Initial Presentation of Inguinal Hernia, Journal of Surgical Case Reports. (2014) 2014, no. 6, 1653–1655, 10.1093/jscr/rju056. PMC4040867 24887429

[8] Amoah M. , et al.Spontaneous Scrotal Enterocutaneous Fistula in a 65-Year-Old Man, Ghana Medical Journal. (2011) 45, no. 3, 124–126.

[9] Arogundade A. , Ajape A. A. , and Adesina M. D. , et al.Spontaneous Scrotal Faecal Fistula in a Nigerian Adult: Review of Literature and Proposal for Management Protocol, East African Medical Journal. (2016) 93, no. 10, 544–548.

[10] Arora B. , Scrotal Fecal Fistula due to Enteric Perforation in Inguinal Hernia, International Journal of Case Reports and Images. (2016) 7, no. 12, 791–794.

[11] Sohrabi C. , Mathew G. , Maria N. , Kerwan A. , Franchi T. , and Agha R. A. , The SCARE 2023 Guideline: Updating Consensus Surgical CAse REport (SCARE) Guidelines, International Journal of Surgery. (2023) 109, no. 5, 1136–1140, 10.1097/JS9.0000000000000373. 37013953 PMC10389401

[12] Townsend C. M. , Beauchamp R. D. , and Evers B. M. , Mattox K. L. , Sabiston Textbook of Surgery, 2021, Elsevier.

[13] Jha R. , Shrestha S. , Bhatta B. R. , Upadhayay R. P. , and Prasad R. , Straining Induced Spontaneous Bowel Transection in a Patient With Incarcerated Inguinal Hernia With Cryptorchidism: A Case Report, International Journal of Surgery Case Reports. (2025) 128, 10.1016/j.ijscr.2025.111093, 111093. 40015228 PMC11910128

[14] Moloney G. E. , Results of Nylon Darn Repair of Hernia, Lancet. (1958) 1, no. 7015, 273–278. 13515182 10.1016/s0140-6736(58)91027-4

